# Nanoceria Inhibit the Development and Promote the Regression of Pathologic Retinal Neovascularization in the *Vldlr* Knockout Mouse

**DOI:** 10.1371/journal.pone.0016733

**Published:** 2011-02-22

**Authors:** Xiaohong Zhou, Lily L. Wong, Ajay S. Karakoti, Sudipta Seal, James F. McGinnis

**Affiliations:** 1 Department of Ophthalmology, University of Oklahoma, College of Medicine, Dean McGee Eye Institute, Oklahoma City, Oklahoma, United States of America; 2 Advanced Materials Processing Analysis Center, Mechanical Materials Aerospace Engineering, Nanoscience, and Technology Center, University of Central Florida, Orlando, Florida, United States of America; 3 Department of Ophthalmology and Cell Biology, Oklahoma Center for Neuroscience, Oklahoma City, Oklahoma, United States of America; University of Bristol, United Kingdom

## Abstract

Many neurodegenerative diseases are known to occur and progress because of oxidative stress, the presence of reactive oxygen species (ROS) in excess of the cellular defensive capabilities. Age related macular degeneration (AMD), diabetic retinopathy (DR) and inherited retinal degeneration share oxidative stress as a common node upstream of the blinding effects of these diseases. Knockout of the *Vldlr* gene results in a mouse that develops intraretinal and subretinal neovascular lesions within the first month of age and is an excellent model for a form of AMD called retinal angiomatous proliferation (RAP). Cerium oxide nanoparticles (nanoceria) catalytically scavenge ROS by mimicking the activities of superoxide dismutase and catalase. A single intravitreal injection of nanoceria into the *Vldlr-/-* eye was shown to inhibit: the rise in ROS in the *Vldlr-/-* retina, increases in vascular endothelial growth factor (VEGF) in the photoreceptor layer, and the formation of intraretinal and subretinal neovascular lesions. Of more therapeutic interest, injection of nanoceria into older mice (postnatal day 28) resulted in the regression of existing vascular lesions indicating that the pathologic neovessels require the continual production of excessive ROS. Our data demonstrate the unique ability of nanoceria to prevent downstream effects of oxidative stress in vivo and support their therapeutic potential for treatment of neurodegenerative diseases such as AMD and DR.

## Introduction

Mammalian cells produce cellular energy in mitochondria by using oxygen to metabolize molecular substrates. The vast majority(∼98%) of the products of this oxidative metabolism are beneficial while about 2% are highly toxic compounds such as singlet oxygen, the hydroxide ion, hydrogen peroxide, etc.[1]. These ROS [2], [3] can react with and damage almost any type of molecule within the cell including proteins, DNA, RNA and lipids [4]. In addition to mitochondria, nicotinamide adenine dinucleotide phosphate (NADPH) oxidase, and nitric oxide synthase contribute to the production of intracellular ROS, and reactive nitrous oxide species, respectively [5]. To maintain redox balance, mammalian cells posses endogenous antioxidant defenses that include catalytic proteins such as superoxide dismutase, catalase [6], heme-oxygenase [7], and thioredoxin [8] as well as small molecules such as glutathione, NADPH, etc [9]. Oxidative stress occurs when the level of ROS exceeds the ability of the cells' antioxidant defenses to scavenge or destroy them [10]–[13]. Being constantly bombarded with photons of light, and possessing the highest rate of oxygen metabolism, the retina is therefore at higher risk of oxidative damage due to redox imbalance.

Many neurodegenerative diseases result in the programmed death of neurons. These include illnesses which are known to be inherited such as Huntington Disease [14] and retinitis pigmentosa [15], [16] as well as many others that may be environmentally induced or are of questionable origin, such as Parkinson Disease [17], Alzheimer Disease [18] and AMD [19]. Interestingly, all of these diseases are thought to share a chronic or acute rise in ROS as a common node between the primary cause and neuronal degeneration. Strong evidence that oxidative damage is a major contributor to the disease progression of AMD, DR, and glaucoma is accumulating [20]–[22]. In addition to retinal degeneration, chronic inflammation and vascular defects are also observed in some of these blinding diseases. Currently, the relationship between oxidative stress, or oxidative damage, to the manifestation of these disease phenotypes is still unclear. Recent studies show that rise in ROS activates the signal transducers and activators of transcription 3 (STAT3) pathway and upregulates retinal vascular endothelial growth factor (VEGF), an angiogenic protein, to cause abnormal blood vessel growth [23]. We hypothesize that the chronic rise of ROS is an “Achilles' Heel” for AMD and other degenerative diseases and that by targeting excess ROS for destruction, the downstream damage and disease symptoms can be prevented and/or decreased. To test this hypothesis, we choose the *Vldlr-/-* mouse, a model for a form of AMD known as retinal angiomatous proliferation (RAP), to investigate the relationship between oxidative damage and retinal neovascularization (RNV). This mouse carries a loss-of-function mutation in the *very low density lipoprotein receptor* gene (*Vldlr*) [24]. We [25] and others [26]–[28] have shown that the retina of the *Vldlr-/-* mouse has phenotypic characteristics similar to those of RAP patients. Previous studies show that new blood vessels sprout from the inner retina of these mice as early as postnatal day (P) 16 [29]. Intra-, and subretinal vascular lesions are well established by 5 weeks of age and a rise in retinal VEGF correlates with the neovascularization phenotype [25], [27], [29]. Retinal focal scarring due to engulfment of vascular lesions by retinal pigment epithelial (RPE) cells further remodels the retinal architecture. By 4 months of age, cone and rod dysfunctions are apparent [27], [30]. In this study, we focus on the characterization of the early events of retinal defects (P10-P35), specifically the temporal development of RNV and retinal VEGF expression, the effects of a potent antioxidant on prevention of RNV formation, and regression of existing RNV.

Cerium oxides, because of their redox capacity, are excellent oxygen buffers and when synthesized as nanoparticles (3–5 nm diameter) exhibit increased oxygen vacancies and can regenerate their activity to catalytically scavenge ROS [31]. Thoroughly characterized 3–5 nm (individual crystallites) nanoceria were used in the present experiments. An extensive characterization ensures the abundance of catalytically active Ce^3+^ oxidation state and stable aqueous dispersion. (Detailed characterization is reported in Text S1, and Fig. S1.)

We previously demonstrated [32] that injection of nanoceria into the vitreous, prevents increases in retinal ROS, light damage and blindness in albino rats. In this study, using assays that detect ROS and ROS-mediated damage, our data demonstrate that the aberrant developmental increases in ROS and ROS-mediated damage, which occur in the *Vldlr-/-* retina, are inhibited by a single intravitreal injection of nanoceria. The data also demonstrate that the nanoceria prevent the rise in retinal VEGF, the development of vascular lesions in the photoreceptor cell layer of the retina (neovascular blebs) and the appearance of subretinal neovascular tufts. We further show that the nanoceria cause the regression of all angiogenic characteristics of the *Vldlr-/-* retina even when they are injected after the mutant retinal phenotypes are already established. These data support our general hypothesis and suggest that the nanoceria will be useful as a therapeutic treatment for retinal angiomatous proliferation and other blinding diseases associated with oxidative stress.

## Results

### Pathologic blood vessels in the *Vldlr-/-* retina grow from the inner retina through the avascular ONL and into the subretinal space

**Figure 1:**
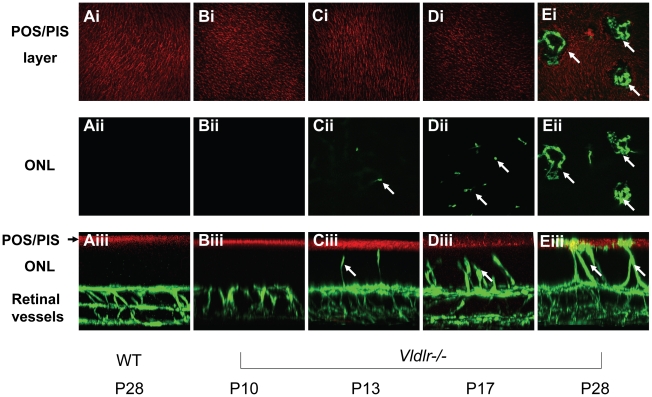
Origin of retinal vascular lesions in the *Vldlr-/-* eye. Confocal microscopy was used to generate “optical sections” through whole mounts of retinas following the vascular filling assay to label all retinal blood vessels (green). The cone sheaths were labeled red with peanut agglutinin to visualize the outer retina. Stacks of the optical sections were assembled to show either the photoreceptor cell outer and inner segment region (POS and PIS; **A1** through **E1**) or the outer nuclear layer (ONL; **A2** through **E2**). The entire stack of sections through the retina was assembled for 3D reconstruction which was then rotated 90° for a cross-sectional view of the retinas at different developmental ages (**A3** through **E3**). See text for further description.

Retinal angiomatous proliferation in humans has been described as having new blood vessels growing from existing ones in the neural retina through the outer nuclear layer (ONL) towards the choroid where they eventually fuse with the choroidal blood supply [33]. The direction of neovascular growth in the *Vldlr-/-* retina was originally shown to be from the inner retina into the subretinal space [28], [29] but this was disputed in a recent paper where the authors concluded that the new vessels actually originate in the choroid [26]. We, therefore, undertook a developmental study on new blood vessel growth using a vascular filling assay and confocal imaging, which enabled us to distinguish between these alternative conclusions. The vascular network was labeled with fluorescein-Dextran (green), and the cone outer segments were labeled with peanut agglutinin (red). The whole retina was then mounted with the photoreceptors facing up and the ganglion cell layer down. Optical sections were taken every 0.2 µm from the top down. This enabled defined stacks of sections to be visualized as well as the 3-dimensional stack through the entire retina to be rotated 90°. Representative images from such studies are shown in **Fig. 1**. The cone sheaths form a continuous surface (**Fig. 1, A1–E1**) in the P28 wild type (WT) C57 retina and in the *Vldlr-/-* retinas at P10, P13 and P17. However, at P28 this continuity was disrupted by pathologic blood vessels. Looking down at the stacked images at the level of the ONL (**Fig. 1, A2–E2**), we did not observe vessels in the P28 WT nor in the P10 *Vldlr-/-*. Pathologic vessels first appeared as dots in the *Vldlr-/-* P13 ONL (**Fig 1, C2**) and progressively increased in number through P17 (**Fig 1, D2)** and then coalesced by P28 (**Fig 1, E2**). By rotating the entire stack of optical sections 90° (**Fig 1, A3–E3**), we created a cross-sectional view where the cones are on the top and the inner retina at the bottom. In the *Vldlr-/-* retina, pathologic vessels were seen to grow from the inner retina (**Fig 1, C3**) into the ONL in increasing numbers (**Fig 1, D3**), until they coalesced and pushed through the cone outer segments (**Fig 1, E3**) to localize at the subretinal space in contact with the RPE. These data definitively demonstrate that pathologic new vessels in the *Vldlr-/-* mouse grow from the inner retina towards the avascular zone in the ONL and the RPE and not in the reverse direction. We further demonstrate that new vessel growth commences as early as P13 before eye opening.

### Indicators of oxidative stress are up in *Vldlr-/-* retinas and go down with nanoceria treatment

**Figure 2:**
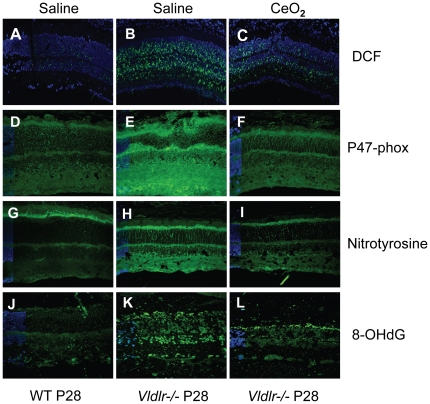
Nanoceria reduce oxidative stress in the *Vldlr-/-* retina. Retinal sections from saline injected WT mice (**A,D,G,J**); saline injected *Vldlr-/-* mice (**B,E,H,K**) and CeO_2_ injected (**C,F,I,L**) *Vldlr-/-* mice are shown as imaged by confocal microscopy. The DCF assay (**A,B,C**) visualizes ROS as punctuate fluorescence and demonstrates a very low level of ROS in the normal (**A**), a considerable amount in the *Vldlr-/-* (**B**), and a greatly reduced amount in the retina of the *Vldlr-/-* mice injected with CeO_2_ (**C**). Similar results were obtained with the other three assays. NADPH-oxidase, (P47-phox; **D,E,F**) a major producer of ROS, was very high in the *Vldlr-/-* retina and almost reduced to control levels in the CeO_2_ injected mice. Nitrotyrosine, (**G,H,I**) a reflection of oxidative activity due to increases in nitric oxide concentration, was highest in the *Vldlr-/-* retina and significantly reduced in the nanoceria injected mice. ROS-mediated damage to DNA was indicated by the labeling of the retina with an antibody against a DNA adduct, 8-hydroxy-2′-deoxyguanosine (8-OHdG; **J,K,L**) which showed little labeling in the control, significant labeling in the saline injected *Vldlr-/-* retina, and a greatly reduced amount in the nanoceria treated retina. DAPI (blue) was used to visualize the nuclei.

To test our hypothesis, we verified that oxidative stress was detected in the newly matured (P28) retina of the *Vldlr-/-* mouse. Initially, we used the 2′,7′-dichloro-dihydro-fluorescein-diacetate (DCF) assay to detect the level of cellular ROS in cryostat sections of retinas from normal and *Vldlr-/-* mice which had been injected intravitreally with either saline or nanoceria on P7. We observed a low level of DCF signal in the WT retina (**Fig. 2A**) but very substantial amounts in the *Vldlr-/-* retina (**Fig. 2B**). However, injection of the nanoceria greatly reduced the level of ROS in the *Vldlr-/-* retina (**Fig. 2C**). We next examined the level of the activating subunit of NADPH oxidase (P-47) in retinal sections (**Fig. 2D-F**). Upregulation of this subunit correlates with an increase in ROS level [34], [35]. We observed a low level of labeling throughout the WT retina (**Fig. 2D**). The *Vldlr-/-* retina showed intense staining in all retinal layers (**Fig. 2E**) whereas the intensity of staining in the retina of the nanoceria-injected *Vldlr-/-* mouse was reduced almost to the control level (**Fig. 2F**). As levels of nitrous oxide species rise within the cell, the nitrosylation of tyrosine residues in proteins increases. We determined that the level of nitrosylation in the *Vldlr-/-* retina had increased using anti-nitrotyrosine. We observed a low level of labeling in the WT retina (**Fig. 2G**), but a very intense labeling in the *Vldlr-/-* retina (**Fig. 2H**). Similar to the previous observation, the level decreased to the control level in the nanoceria-injected (**Fig. 2I**) *Vldlr-/-* mouse. Oxidative damage to DNA results in the production of 8-hydroxydeoxyguanosine (8-OHdG) and can be detected by immunocytochemistry. WT retina (**Fig. 2J**) showed detectable levels of 8-OHdG and the *Vldlr-/-* retina (**Fig. 2K**) had much higher levels. Treatment with the nanoceria reduced the amount of 8-OHdG in the *Vldlr-/-* retina (**Fig. 2L**) to levels similar to the WT control. The dihydroethidine assay (Fig S2) for detection of superoxide anions did not show a difference between the WT controls and the *Vldlr-/-* mice (both were uninjected). Each of the other four independent assays showed an increase in oxidative stress in the *Vldlr-/-* retina compared to the WT control and demonstrated a decrease in oxidative stress in the retinas of *Vldlr-/-* mice injected with nanoceria. Collectively, these data support our hypothesis that oxidative damage increases in the mouse retina as a result of the knockout of the *Vldlr* gene and that the presence of nanoceria inhibits those increases.

### Nanoceria inhibit the aberrant developmental increase of retinal VEGF

**Figure 3:**
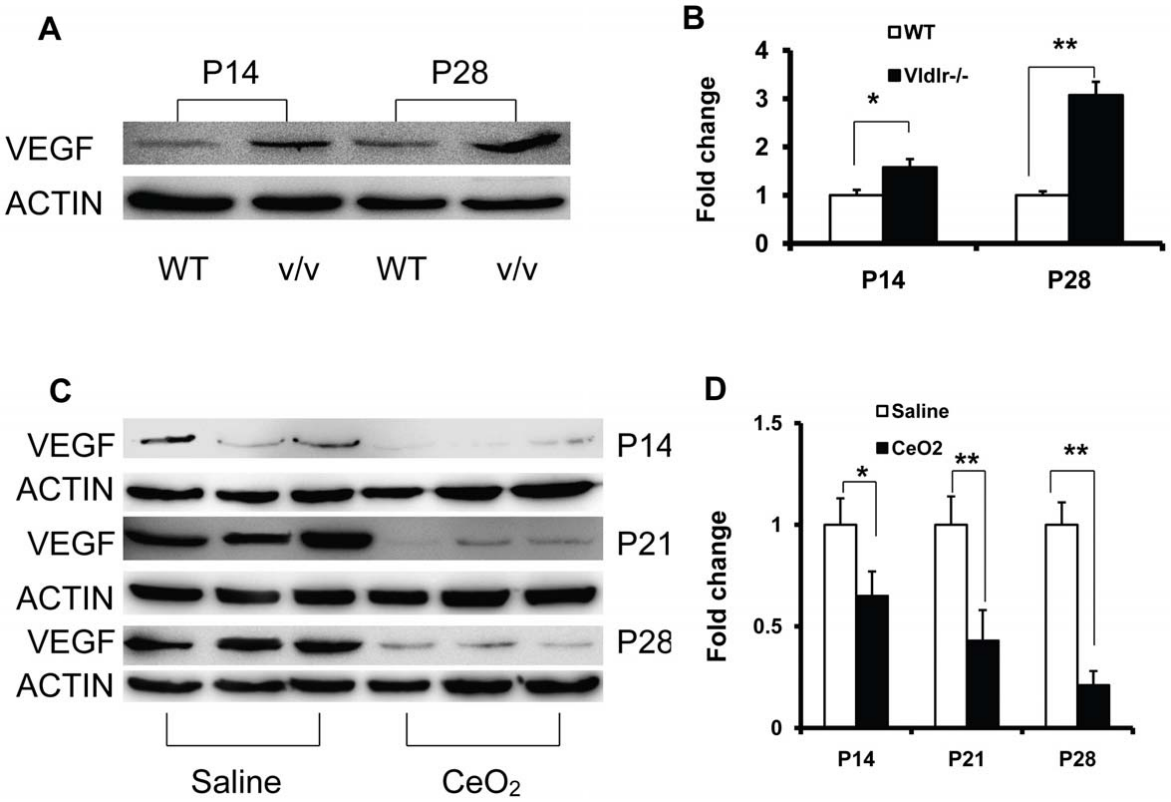
Nanoceria prevent the ectopic increase of VEGF during postnatal development of the *Vldlr-/-* retina. (**A**) Western immunoblots show that VEGF levels were higher in the *Vldlr-/-* retinas than in the WT at P14 and P28. Densitometry of these bands (**B**) indicated that by P28 the *Vldlr-/-* retina had about 3 fold more VEGF than the WT retina. Ectopic developmental increases (P14, P21, P28) of VEGF in the *Vldlr-/-* retina were seen in the immunoblots (**C**) of the retinas of saline injected *Vldlr-/-* mice, and significant reduction of VEGF was observed in retinas of mice injected with nanoceria at P7. Densitometric analysis (**D**) of the bands showed about a five-fold decrease in VEGF level by P28 following injection of nanoceria at P7.

Previous studies show that elevated retinal *vegf* mRNA can be detected at P14 [27]. We show here that upregulation of retinal VEGF protein level can be detected as early as P14 in the *Vldlr-/-* mice (**Fig. 3A,B**). By Western blot analysis, we demonstrated that VEGF was at least 3 fold higher in the *Vldlr-/-* retina compared to the WT at P28 (**Fig. 3B**). Injection of a single dose of nanoceria at P7 resulted in a progressive decrease in VEGF levels in the *Vldlr-/-* retina. By P28, the amount was about 5 fold less than that in the saline injected eyes (**Fig 3C,D**). These data are consistent with the interpretation that the scavenging of ROS and the inhibition of oxidative damage by the nanoceria, inhibit the upregulation of VEGF in the *Vldlr-/-* retina.

**Figure 4:**
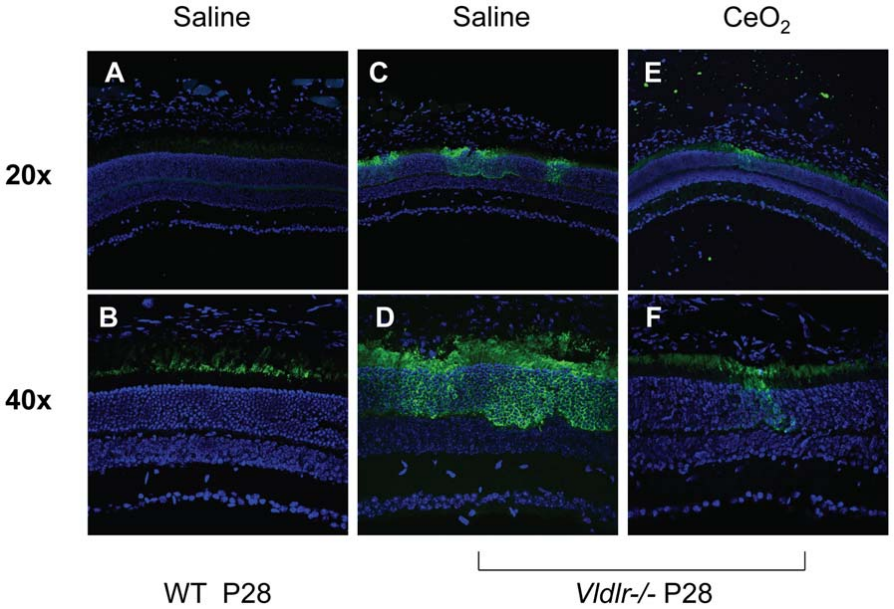
Nanoceria inhibit the ectopic expression of VEGF in the ONL of the *Vldlr-/-* retina. Photomicrographs of the *Vldlr-/-* retina showed discontinuous heavy staining of VEGF across the ONL (**C,D**) at P28. The labeling was greatly reduced in the nanoceria injected (**E,F**) *Vldlr-/-* mice. WT retinas (**A,B**) had low levels of VEGF in the rod outer segments. DAPI (blue) was used to visualize the nuclei.

In parallel experiments, we examined the localization of VEGF in retinal sections. The WT retinas (**Fig. 4A**) showed very little labeling of VEGF. At higher magnification (**Fig. 4B**), VEGF was seen to be primarily localized to the region of the outer segments of photoreceptor cells. However, the pattern of labeling in the *Vldlr-/-* retina showed intense discontinuous fluorescence across the retina (**Fig. 4C,D**) which was restricted to the photoreceptor cell layer. The labeling was immediately adjacent to vascular lesions and progressively diminished as the distance from the lesion increased. At higher magnification (**4D**), the area of intense labeling showed that all of the cellular compartments of the photoreceptors adjacent to the lesions were labeled. The corresponding age-matched *Vldlr-/-* mice, which had received an intravitreal injection (172 ng) of nanoceria on P7, showed many fewer regions of labeling and within those, the intensity was significantly decreased (**Fig. 4E,F**). These data demonstrate that photoreceptor cells at or near lesion sites in the *Vldlr-/-* retinas have high concentrations of VEGF and that a single injection of the nanoceria at P7 inhibits the developmental increase in retinal VEGF for at least three weeks. These data support our hypothesis that the rise in retinal VEGF in the *Vldlr-/-* retina is due to excess ROS and can be prevented by the scavenging activities of nanoceria.

### Nanoceria inhibit development of intraretinal neovascular blebs and subretinal neovascular tufts

**Figure 5:**
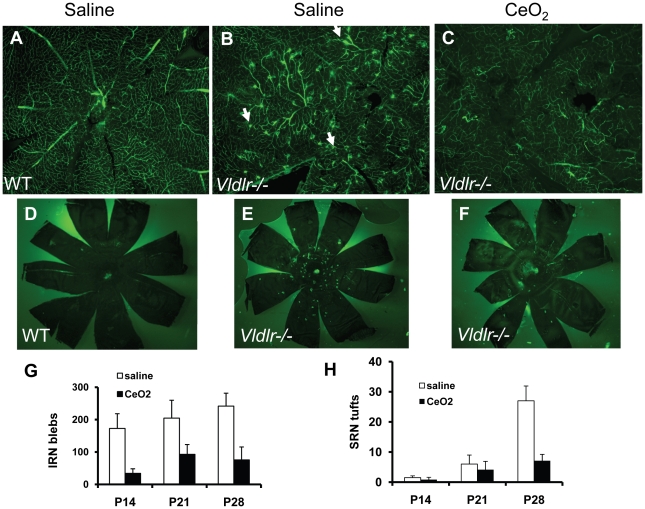
Nanoceria inhibit the development of pathologic intra-, and sub-retinal vascular lesions in the *Vldlr-/-* retina. Photomicrographs of whole mount retinas (**A–C**) and eyecups (RPE, choroid, and sclera) (**D–F**) from P28 animals are shown. All retinal blood vessels were labeled green by the vascular filling assay. WT retinas (**A**) showed the normal web-like retinal vasculature whereas those from the *Vldlr-/-* mice (**B**) showed numerous intraretinal vascular lesions or “blebs” (IRN blebs). See white arrows for examples. A single injection of nanoceria at P7 inhibited (**C**) the appearance of these lesions. Eyecups from WT mice (**D**) showed no SRN “tufts” but those from *Vldlr-/-* mice (**E**) had many bright SRN tufts. A single injection of nanoceria on P7 inhibited the appearance of these SRN tufts (**F**). **G** and **H** show the quantitative analyses of IRN blebs and SRN tufts from this set of the experiment. Data were from nine animals, three at each of the three developmental ages (P14, P21, P28) with or without nanoceria. * p<0.05; **p<0.01. See text for further information.

The rodent and human retinas have two blood supplies, the retinal and the choroidal vasculature. The *Vldlr-/-* retina develops retinal neovascularization initially, followed by choroidal neovascularization at a later age (current study and [29]). The retinal vasculature of the *Vldlr-/-* mouse exhibits a developmental increase in “neo” vessels which are absent from control retinas (**Fig. 1**) suggesting the possibility that these vessels arise as a result of the increase in retinal VEGF and that the nanoceria can inhibit their development. To test this, we performed the vascular filling assay to determine if a single intravitreal injection of the nanoceria on P7 could inhibit the formation of these pathologic blood vessels when visualized on P28. Representative whole mount retinal vasculature images are shown in **Fig. 5A–C**. With this assay, the larger vessels found in the superficial layer showed intense labeling whereas the smaller vessels found in the deeper layers exhibited a less intensely labeled meshwork (**Fig. 5A**). Because the retinal vasculature is confined to the inner retina (from the neural fiber layer to the outer plexiform layer) of the WT animal, most of the blood vessels could be brought into sharp focus using a 10X objective. Injection of saline has no effect on the normal retinal vasculature (**Fig. 5A**). On the contrary, we observed many brightly labeled vessels that were either coiled or enlarged in the saline injected *Vldlr-/-* animal (**Fig. 5B**). Because these neovessels were small, irregular in shape, and were found throughout the whole retina (most of them in the ONL), we called these intraretinal neovascular blebs (IRN blebs). Because these blebs were located in different focal planes within the retina, many of them appeared as out-of-focus blur in the representative image (**Fig. 5B**). However, in the nanoceria injected *Vldlr-/-* eyes (**Fig. 5C**), we observed a substantial reduction of these neovessels.

The vascular filling assay also enables visualization of subretinal neovascular lesions that have penetrated through the retina to be in contact with the RPE in the subretinal space. Eyecup flat mounts containing the RPE-choroid-sclera are “pie-cut” and placed with the RPE facing up (**Fig. 5D–F**). The pigmented RPE prevented visualization of any of the choroidal vasculature in the normal C57 mouse (**Fig. 5D**) but the eyecups from the saline injected *Vldlr-/-* mice (**Fig. 5E**) had many bright subretinal neovascular (SRN) tufts which projected into the RPE layer. However, the eyes of the *Vldlr-/-* mice that had been injected with nanoceria (**Fig. 5F**) had far fewer SRN tufts. Because the IRN blebs and the SRN tufts in the *Vldlr-/-* eyes are readily visible as distinct spots, they can be quantified. Enumeration of the IRN blebs (**Fig. 5B**) in the *Vldlr-/-* retinas showed a progressive increase in their number from P14 through P28 and a reduction (**Fig. 5G**) at each of the ages following a single injection of nanoceria at P7. Likewise, the SRN tufts (**Fig. 5E**) increased about six-fold (**Fig. 5H**) from P14 to P28 in the saline injected eyes whereas the nanoceria injected eyes (**Fig. 5H**) had four-fold fewer SRN tufts at P28 than were detected in the saline injected eyes. Collectively, these data demonstrated that the pathologic angiogenesis in the *Vldlr-/-* eye can be inhibited by a single injection of the nanoceria and that the extent of inhibition can be quantified.

### Nanoceria cause down regulation of VEGF and regression of existing pathologic neovessels

**Figure 6:**
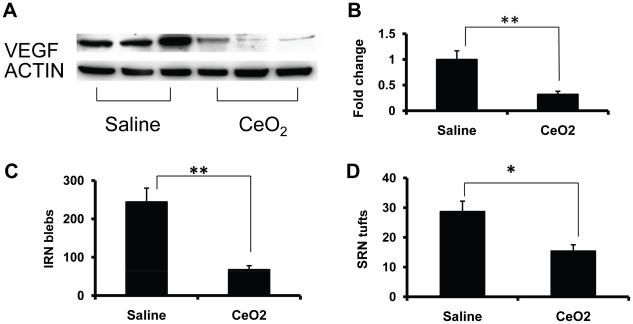
Retinal vascular lesions in the *Vldlr-/-* retinas require continual production of excess ROS. *Vldlr-/-* mice were injected at P28 with saline or nanoceria and killed one week later on P35. Analysis of VEGF levels by Western blots (**A**) showed a four-fold reduction (**B**) within one week of nanoceria injection. The numbers of IRN blebs (**C**), and SRN tufts (**D**) were also dramatically reduced. * p<0.05; **p<0.01.

Injection of nanoceria at P7 dramatically reduced the developmental increase of retinal VEGF (**Fig. 3**), IRN blebs and SRN tufts (**Fig. 5**) when analyzed at P28. We next asked whether developmental changes that had occurred prior to injection of nanoceria could be reversed. For this experimental paradigm (**Fig. 6**), *Vldlr-/-* mice received an intravitreal injection of nanoceria at P28 and the eyes were analyzed a week later at P35. The results were striking in that the presence of the nanoceria for only one week caused down regulation of VEGF (**Fig. 6A, B**), and regression of both IRN blebs (**Fig. 6C**), and SRN tufts (**Fig. 6D**) to levels similar to those which resulted from the presence of nanoceria for the three-week period from P7 to P28. We conclude from these data that the continued presence of elevated levels of VEGF and maintenance of the pathologic retinal neovessels, require the continual production of excess ROS and that their inhibition by nanoceria produces a rapid decrease in each parameter.

## Discussion

### Oxidative stress is proangiogenic

Our results support a causative relationship between oxidative stress and the pathologic new blood vessel formation in the *Vldlr-/-* retina. Using biomarkers for oxidative damage (Fig. 2), we definitively showed that oxidative damage was apparent in the retinas of *Vldlr-/-* mice by P28. We also demonstrated that we could dramatically reduce the level of oxidative damage in the retina (Fig. 2), the abnormal developmental rise of VEGF, a potent angiogenic factor, in the ONL (Fig. 3–4), and the growth of pathologic retinal new blood vessels by a single application of nanoceria, a catalytic and regenerative antioxidant (Fig. 5) in the vitreous of the *Vldlr-/-* mouse. Our results showed that reduction of oxidative stress in the young retinas of *Vldlr-/-* mice reduced retinal neovascularization. Our findings are consistent with the observations in another mouse model that develops retinal neovascularization. Superoxide dismutase 1 (Sod1), a major antioxidant enzyme that neutralizes superoxides in the cytosol of cells, when deficient in the mouse, causes oxidative damage in the retina [36]. Dong and coworkers [37] showed that the *sod1-/-* deletion, when in a genetic background overexpressing VEGF in the rod photoreceptors (Tg-rhoVegf), produced significantly higher numbers of subretinal neovascular lesions, indicating that oxidative stress is proangiogenic. Collectively, this evidence suggests that oxidative stress may also be proangiogenic in eye diseases such as AMD and DR.

### Regenerative nanoceria have prolonged antioxidant and anti-angiogenic effects

In this study, we also demonstrated that the nanoceria possessed radical scavenging activity in vivo. The amount of ROS, measured by DCF fluorescence, in the *Vldlr-/-* retina remained at a reduced level three weeks after nanoceria application (Fig. 2). The regenerative property of nanoceria demonstrated in vitro in suspension [31] appears to be retained when applied in biological environments, more specifically in the retina. Besides decreased ROS, we observed significant reduction of intra- and subretinal NV using the experimental paradigm of injecting nanoceria at P7 and analyzing the anti-angiogenic effects at P28. This prolonged anti-angiogenic effect exhibited by the nanoceria is a substantial improvement over the transient anti-angiogenic effect demonstrated by Dorrell and coworkers in the *Vldlr-/-* mouse [27]. They used a cocktail of Macugen, an integrin antagonist, and a fragment of tryptophan transfer RNA synthetase (T2-TrpRs) with angiostatic activity, in young *Vldlr-/-* mouse. The anti-angiogenic effect was observed during 8 days of treatment (P12 injection and P20 analysis) but this effect was abolished within 2–3 weeks of injection, most likely due to clearance of the agents.

In another experimental paradigm, we administered nanoceria at P28, when retinal neovascularization was well underway, and examined the retinal vasculature one week later at P35. We observed dramatic reductions of VEGF levels and intra- and sub-retinal neovascular lesions (Fig 6). These results further support our hypothesis that oxidative stress is proangiogenic and is likely to lie upstream of the pathogenesis of RNV in the *Vldlr-/-* mouse. Furthermore, the sensitivity of new blood vessels to ROS levels may be related to the malformation of these newly formed vessels. Chen and co-workers [30] showed that at six weeks of age, many of the endothelial cells in the subretinal neovasculature of *Vldlr-/-* retinas were not associated with alpha-Smooth Muscle Actin-positive (ASMA-positive) pericytes. The potential lack of pericytes or the association of dysfunctional pericytes with these new blood vessels was further demonstrated by the reduction of the pericyte survival factor, platelet-derived growth factor-BB (PGDF-BB) in the mutant retinas [30]. Because new blood vessels found in patients with proliferative diabetic retinopathy are leaky and do not mature [38], [39], we speculate that application of the nanoceria may be an effective treatment to cause regression of these pathologic new blood vessels.

### VEGF overexpression is in the ONL

In this study, we showed that ectopic VEGF expression in the retinas of *Vldlr-/-* mice was within photoreceptor cells and coincided with zones of intra- and subretinal NV. Since *Vldlr* mRNA is highly expressed in the ONL and the outer segment of photoreceptors in the WT retinas [27], we speculate that the lack of VLDLR renders photoreceptor cells more vulnerable to oxidative stress, and therefore causes upregulation of VEGF. Not surprisingly, the intra- and subretinal NV phenotypes we observed in *Vldlr-/-* mice were very similar to the developmental progression of subretinal neovascularization observed in the Tg-rhoVegf mice [40]. In this transgenic mouse, ectopic VEGF is expressed by rod photoreceptors in the ONL. New vessels sprout from the deep layer of the retinal vasculature, invade the ONL and subretinal space and eventually these neovascular lesions are engulfed by RPE cells. One major difference between these two models is the eventual anastomoses of the subretinal neovascular lesions with the choroidal vasculature in the *Vldlr-/-* mice, which is not observed in the Tg-rhoVegf mice.

Even though anti-VEGF therapy may be an effective treatment for neovascular diseases of the eye, recent findings suggest that a low level of VEGF is normally present in the adult rodent retina, and is necessary to maintain the functions of retinal neurons and glia [41]. Systemic or localized neutralization of VEGF may result in neuronal and glial cell death. We, therefore, propose that therapies using nanoceria may be a superior alternative as they act upstream of the angiogenic pathway and do not interfere with the normal production and functions of VEGF.

## Materials and Methods

### Animals

Breeding pairs of mutant mice with targeted deletion of the *Vldlr* gene (B6;129S7-*Vldlr^tm1Her^*/J; *Vldlr-/-*) were obtained from the Jackson Laboratory (Bar Harbor, ME). C57Bl/6 were used as WT controls.

### Ethics Statement

Animals were cared for and handled according to the Association for Research in Vision and Ophthalmology statement for the use of animals in vision and ophthalmic research. The study was approved by the University of Oklahoma Health Sciences Center Institutional Animal Care and Use Committee (OUHSC IACUC) and the Dean McGee Eye Institute (DMEI) IACUC. The approved protocol numbers were 09-027 and 09-105 from the OUHSC IACUC, and D-09-027 and D-09-105 from the DMEI IACUC.

### Synthesis of nanoceria

Cerium oxide nanoparticles were synthesized using simple wet chemistry methods as described previously [31]. Briefly, stoichiometric amount of cerium nitrate hexahydrate (99.999% from Sigma Aldrich) was dissolved in deionized water. The solution was oxidized using excess of hydrogen peroxide. After the synthesis of nanoparticles, the pH of the solution was maintained below 3.0 using nitric acid (1N) to keep the synthesized nanoceria in suspension.

### Intravitreal injection of nanoceria

Intravitreal injection was performed as described previously [32]. Briefly, mouse pups at P7 were “cold” anesthetized by placement on ice. One µl of 1 mM (172 ng) cerium oxide nanoparticles (nanoceria) in saline was injected into the vitreous under an ophthalmic operating microscope. Control mice were injected with 1 µl of saline. Both eyes of each animal received the same treatment. Animals with retinal bleeding or lens injury following the injection procedure were excluded from the study. Animals were euthanized at 7, 14, or 21 days after injection, and the eyes were rapidly enucleated.

### FITC-dextran vascular filling assay

Angiography was performed in deeply anesthetized mice using intracardial injection of 30 µl, 50 mg/ml fluorescein isothiocyanate-conjugated high molecular weight Dextran (FITC-Dextran, ∼2×10^6^ molecular weight; Sigma) [28]. Eyes were enucleated three minutes after perfusion. They were fixed in 4% paraformaldehyde at room temperature for 2 hours. Cornea, iris and lens were removed from the eyecup and then the retina was carefully dissected free of the rest of the eye. Radial cuts were made in the retinas and eyecups from the edge to the equator for flat-mounting. For retinas, the ganglion cell layer was facing up; for eyecups, the RPE was facing up. Images were captured using a Nikon Eclipse 800 fluorescence microscope equipped with a Micromax CCD camera (Princeton Instruments, Trenton, NJ) and the MetaVue software (Molecular Devices, Downingtown, PA).

### Western blot analysis

Western blot analysis was performed as described previously [42]–[45]. Fifty µg of retinal protein per sample were used for SDS-PAGE mini-gels. After electrophoresis, proteins were transferred to nitrocellulose paper, washed for 2×10 min in TTBS (0.1% Tween 20 in 20 mM Tris-HCl, pH 7.4, and 410 mM NaCl) and blocked with 10% BSA in TTBS with 5% milk for 2 hours at room temperature. Blots were incubated with rabbit anti-VEGF polyclonal antibody (1∶500, Santa Cruz Biotechnology, Santa Cruz, CA) overnight at 4°C; washed three times for 5 min each with TTBS; incubated for 1 hour with anti-rabbit IgG HRP-linked secondary antibodies (1∶3000, GE Healthcare, Pittsburgh, PA); washed four times for 10 min each with TTBS and developed by enhanced chemiluminescence.

### Immunohistochemistry and confocal imaging

Immunohistochemical staining was performed as described previously [42]–[46]. The 4% paraformaldehyde fixed eye was cut along the vertical meridian, and cryostat sections were incubated with rabbit anti-VEGF polyclonal antibody (1∶100, Santa Cruz Biotechnology, Santa Cruz, CA), and then secondary antibody conjugated to fluorescein isothiocyanate (Vector Labs, Burlingame, CA). In control experiments, the primary antibody was incubated for 2 hours at 4°C with its blocking peptides before applying to the sections. Sections were visualized and analyzed with a confocal laser scanning microscope system (IX81-FV500; Olympus, Melville, NY).

Flat mounts of the neural retina, after blocking in PBS with 0.5% Triton X-100, 2% BSA, and 10% goat serum for 30 minutes at room temperature, were stained with biotinylated-peanut agglutinin (1∶200, Vector Labs, Burlingame, CA) overnight at 4°C, followed by incubation with Texas red-streptavidin (1∶1000, Vector Labs, Burlingame, CA) at room temperature for 60 minutes. When examined under confocal microcopy, the cone sheaths in the neural retina appeared red, and the retinal vasculature green. Stacks of images (0.2 µm) spanning the entire thickness of the retinal vasculature were generated and three-dimensional reconstruction of the retinal vasculature was obtained using Fluoview image analysis software (Olympus).

### Reactive oxygen species detection

Reactive oxygen species (ROS) generation was examined by two independent methods [47], [48]. Dichlorofluorescein (DCF), the oxidation product of 5-(and-6)-chloromethyl-2′,7′-dichlorodihydrofluorescein diacetate, acetyl ester (CM-H_2_DCFDA; Invitrogen, Carlsbad, CA) emits a green fluorescent signal localized primarily to mitochondria, and is a marker of cellular oxidation by hydrogen peroxide, hydroxyl radicals and peroxynitrite. Unfixed retinal cryostat sections were incubated with CM-H_2_DCFDA (10 µM) for 60 minutes at 37°C. DCF detection at 505 nm was visualized by confocal microscopy. Dihydroethidium (DHE) is oxidized on reaction with superoxide to ethidium bromide, which binds to DNA in the nucleus and fluoresces red. Serial cryosections from fresh-frozen retinas were incubated with DHE (0.625 µM) (Invitrogen, Carlsbad, CA) at 37°C for 20 minutes, followed by confocal microscopy with detection at 585 nm.

### Immunofluorescence staining for oxidative damage

We performed immunostaining using antibodies against biomarkers of oxidative damage according to previously described methods [49], [50]. The activating subunit for NADPH oxidase, p47phox, was detected with rabbit anti-p47phox (1∶50, Santa Cruz Biotechnology, Santa Cruz, CA). Nitrotyrosine and 8-hydroxy-2-deoxyguanosine (8-OHdG) were detected using rabbit anti-nitrotyrosine (1∶100, Chemicon, Temecula, CA) and goat anti-8-OHdG (1∶150, Chemicon, Temecula, CA), respectively. For anti-nitrotyrosine staining, antigen retrieval was done by covering sections with 0.02 M citrate buffer (pH 6.0) and heating them in a microwave oven for 3 min. After cooling to room temperature, additional buffer was added and the slides were reheated; this heating/cooling process was repeated three times. For anti-8-OHdG staining, tissues were pretreated with 10 µg/ml of proteinase K for 20 minutes. Subsequent procedures were as described above.

### Statistical analysis

Each experiment was performed at least three times. Representative data were shown. For quantitative analyses, sample size is from three animals (six eyes). Values were expressed as means ± SD. Statistical analyses were performed using Student's *t*-test; *P*<0.05 was considered significant.

## Supporting Information

Figure S1 **Raw XPS spectra of nanoceria samples.** The peaks between 875 and 895 eV belong to the Ce 3d_5/2_ while peaks between 895–910 eV correspond to the Ce 3d_3/2_ energy levels. The higher extent of Ce^3+^ oxidation state in nanoceria could be easily seen with contribution from peaks at 880.1±0.5, 885.2±0.3, 900.1±0.5 and 903.5±0.3eV belonging to the Ce3+ oxidation state. Inset shows the high resolution transmission electron micrograph of nanoceria depicting the individual 3–5 nm particle size of nanoceria in an agglomerate of less than 10 nm.
(DOCX)

Figure S2 **Photomicrographs of dihydroethidium (DHE) labeling of retinal sections.** Superoxide production in retinal sections was assayed by using the oxidative fluorescent dye, DHE. DHE is oxidized on reaction with superoxide to ethidium bromide which binds DNA in the nucleus and fluoresces red. There was no discernable difference in the DHE-labeled retinas from *Vldlr-/-* and WT mice. GCL = ganglion cell layer; INL = inner nuclear layer; ONL = outer nuclear layer.
(TIF)

Text S1 Experimental Details and Results for Characterization of Nanoceria.
(DOCX)
