# Supplementary material for: Nanoceria Inhibit the Development and Promote the Regression of Pathologic Retinal Neovascularization in the Vldlr Knockout Mouse
Source: PLoS One. 2011 Feb 22;6(2):e16733. doi: 10.1371/journal.pone.0016733 (PMC3043063; doi:10.1371/journal.pone.0016733)
Supplement: Figure S1 — Raw XPS spectra of nanoceria samples. The peaks between 875 and 895 eV belong to the Ce 3d5/2 while peaks between 895–910 eV correspond to the Ce 3d3/2 energy levels. The higher extent of Ce3+ oxidation state in nanoceria could be easily seen with contribution from peaks at 880.1±0.5, 885.2±0.3, 900.1±0.5 and 903.5±0.3eV belonging to the Ce3+ oxidation state. Inset shows the high resolution transmission electron micrograph of nanoceria depicting the individual 3–5 nm particle size of nanoceria in an agglomerate of less than 10 nm. (DOCX) [file pone.0016733.s001.docx]

**Fig. S1.** Raw XPS spectra of nanoceria samples. The peaks between 875 and 895 eV belong to the Ce 3d_5/2_ while peaks between 895–910 eV correspond to the Ce 3d_3/2_ energy levels. The higher extent of Ce^3+^ oxidation state in nanoceria could be easily seen with contribution from peaks at 880.1 ±0.5, 885.2± 0.3, 900.1± 0.5and 903.5± 0.3eV belonging to the Ce3+ oxidation state. Inset shows the high resolution transmission electron micrograph of nanoceria depicting the individual 3-5nm particle size of nanoceria in an agglomerate of less than 10 nm.
