# Supplementary figures and images for: Nanoceria Inhibit the Development and Promote the Regression of Pathologic Retinal Neovascularization in the Vldlr Knockout Mouse
Source: PLoS One. 2011 Feb 22;6(2):e16733. doi: 10.1371/journal.pone.0016733 (PMC3043063; doi:10.1371/journal.pone.0016733)

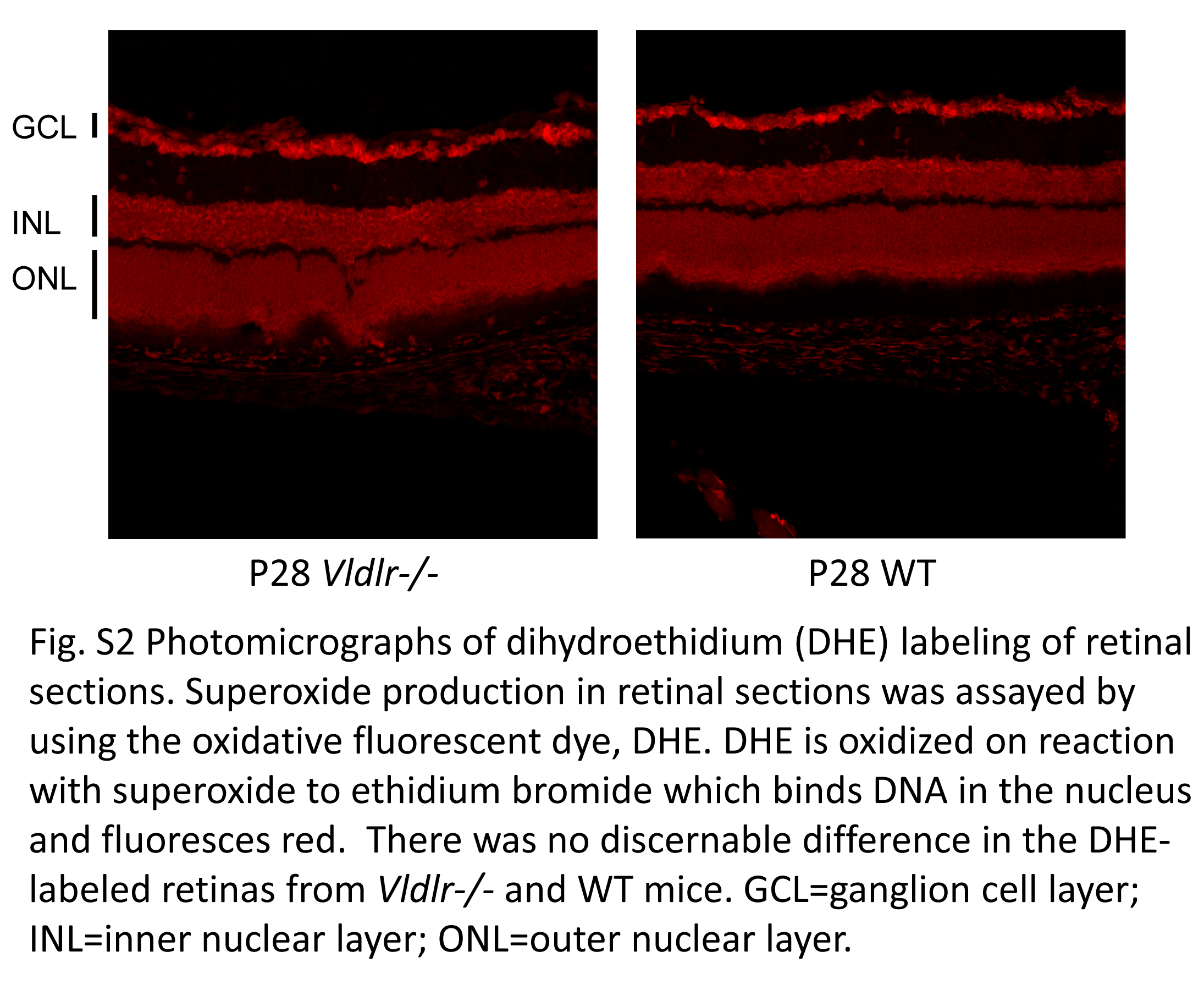

Supplement: Figure S2 — Photomicrographs of dihydroethidium (DHE) labeling of retinal sections. Superoxide production in retinal sections was assayed by using the oxidative fluorescent dye, DHE. DHE is oxidized on reaction with superoxide to ethidium bromide which binds DNA in the nucleus and fluoresces red. There was no discernable difference in the DHE-labeled retinas from Vldlr-/- and WT mice. GCL = ganglion cell layer; INL = inner nuclear layer; ONL = outer nuclear layer. (TIF) [file pone.0016733.s002.tif]
