# Supplementary material for: Nanoceria Inhibit the Development and Promote the Regression of Pathologic Retinal Neovascularization in the Vldlr Knockout Mouse
Source: PLoS One. 2011 Feb 22;6(2):e16733. doi: 10.1371/journal.pone.0016733 (PMC3043063; doi:10.1371/journal.pone.0016733)
Supplement: Text S1 — Experimental Details and Results for Characterization of Nanoceria. (DOCX) [file pone.0016733.s003.docx]

**Supporting Information**

**Text S1: Experimental Details and Results for Characterization of Nanoceria**

1. **Transmission Electron Microscopy:** The particle size and shape of as synthesized nanoceria was analyzed using FEI Tecnai F30 high resolution transmission electron microscope (HRTEM) operated at 300KV with a point to point resolution of 0.2 nm. The samples for TEM were prepared by drop coating the suspension of cerium oxide nanoparticles on a holey carbon coated copper grids followed by drying in vacuum for 24 hrs.
2. **Dynamic Light Scattering:** Zeta potential of the as synthesized cerium oxide nanoparticles was measured using Malvern Nano ZS dynamic light scattering instrument. The zeta potential was measured at a concentration of 5mM and the sample was at first filtered using a 200 nm filter before the measurement to remove any impurities in the solution. The results were repeated in triplicate with 30 measurements in each run. The zeta potential of nanoparticles immediately after diluting in saline was also measured. The table below describes the general observations:

| **Concentration** | **Average Particle Size (nm)** | **Zeta Potential (mV)** |
| --- | --- | --- |
| As synthesized NPs 5mM | 57.7 | +40 ±7 |
| 1mM in saline | 75.7 and 294.2 | +10 ±3 |

The reduction of zeta potential in high concentration salt is a common feature for nanoparticles that are not capped with surface protective and sterically stabilizing groups. Even though the zeta potential of the nanoparticles showed a reduction in magnitude, no apparent precipitation of particles was observed. The particle size measurements showed two different families of aggregates probably due to aggregation of particles owing to reduction in zeta potential. However, this agglomeration is weak agglomeration and the particles still remain in sub nano meter range. The pictures of nanoparticle suspension before and after dilution are shown below and the images confirm that particles were stable before injection.

1. **X-ray Photoelectron Spectroscopy**: The oxidation state of cerium in nanoceria was estimated by XPS analysis of the ceria samples using PHI ESCA 5400 spectrophotometer operated at 300W at a base pressure of 2 x 10^-8^ torr or less and Al Kα as source of x-rays. The samples for the XPS analysis were prepared in glove box with continuous flow of argon by drop coating a 5mM suspension of nanoceria on silicon wafer. The nanoceria coated silicon wafer was transferred to the XPS chamber without any exposure to ambient atmosphere by using a sealed sample transfer chamber. The spectrophotometer was calibrated using a gold standard prior to XPS run and any charge shift in the samples was corrected using adventitious carbon as a reference at binding energy of 284.8 eV. Results are presented in Fig. S1.
2. **Results from Characterization of Nanoceria** As synthesized nanoceria appear transparent and very well dispersed. The measured zeta potential of the nanoceria particles was 40mV± 7 which demonstrates high electrostatic stability of the suspension in as prepared media. The particle size and zeta potential showed a shift in accordance with the usual observation of addition of salt solutions to nanoparticles without any surface capping agent. However, the aggregation does not change the particle size of the cerium oxide nanoparticles. The nanoceria prepared using the room temperature wet chemical synthesis showed particle size in the range of 3-5 nm (inset Fig. S1) and an agglomerate size of around 25 nm from TEM. It has been shown previously that the oxidation state of cerium in nanoceria increases as the particle size decreases [Deshpande et al Applied Physics Letters 2007]. The smaller particle size helps in retaining as high as 30% (from XPS measurements) Ce^3+^ in the nanoceria making it highly catalytically active. Figure S1 shows the XPS spectrum of the nanoceria prepared by drop coating nanoceria on silicon wafer for analysis. The complex XPS spectrum from cerium is split into Ce3d_3/2_ and Ce3d_5/2_ with multiple shake-up and shake-down satellites. The peaks between 875 and 895 eV belong to the Ce 3d_5/2_ while peaks between 895–910 eV correspond to the Ce 3d_3/2_ levels [P. Burroughs et al JCS Dalton, 1976, **17**, 1686] and the peak at 916 eV is a characteristic satellite peak indicating the presence of cerium (IV). To estimate the concentration of trivalent cerium, main peaks from only the 3d_5/2_ portion of the spectrum were used for the peak fit (Peakfit Version 3.0). 3d_5/2_ portion of each spectrum was fitted and the ratio of Ce^3+^ to Ce^4+^ was calculated from the integrated area under the peak.
